# Development and validation of a novel food exchange system for Chinese pregnant women

**DOI:** 10.1186/s12937-023-00902-4

**Published:** 2023-12-01

**Authors:** Ye Ding, Genyuan Li, Man Zhang, Yingying Shao, Jieshu Wu, Zhixu Wang

**Affiliations:** 1 Department of Maternal, Child and Adolescent Health, School of Public Health, Nanjing Medical University, Nanjing, China; 2 Department of Nutrition and Food Hygiene, School of Public Health, Peking University, Beijing, China

**Keywords:** Chinese pregnant women, Food exchange system, Nutrients, Relative validity, Recipe

## Abstract

**Background:**

The dietary nutritional status of pregnant women is critical for maintaining the health of both mothers and infants. Food exchange systems have been employed in the nutritional guidance of patients in China, although their application in the dietary guidance of healthy pregnant women is quite limited. This study aimed to develop a novel food exchange system for Chinese pregnant women (NFES-CPW) and evaluate the relative validation of its application.

**Methods:**

NFES-CPW covers approximately 500 types of food from ten categories and has more elaborate food portion sizes. It established a recommendation index for guiding food selection and used energy, water content, and protein as the exchange basis to balance the supply of energy and important nutrients throughout pregnancy. Furthermore, dietitians used the NFES-CPW and traditional food exchange system to generate new recipes based on the sample recipe. There were 40 derived recipes for each of the two food exchange methods. The food consumption, energy, and key nutrients of each recipe were calculated, and the differences between the two food exchange systems were compared using the Wilcoxon rank sum test or the Chi-square test.

**Results:**

The results revealed that compared to those derived from traditional food exchange system, the NFES-CPW derived recipes had a better dietary structure, as evidenced by the intakes of whole-grain cereals, beans excluding soybeans, potatoes, fruits, fish, shrimp and shellfish, as well as eggs (*P* < 0.05), which were more conducive to reaching the recommended range of balanced dietary pagoda. After calculating energy and nutrients, although these two food exchange systems have similar effects on the dietary energy and macronutrient intake of pregnant women, the intake of micronutrients in NFES-CPW derived recipes was significantly higher than that from the traditional food exchange system, which was more conducive to meeting the dietary requirements of pregnant women. The outstanding improvement are primarily vitamin A, vitamin B_2_, folic acid, vitamin B_12_, vitamin C, calcium, iron, and iodine (*P* < 0.05). Moreover, when compared to recipes obtained from the traditional food exchange system, the error ranges of energy and most nutrients were significantly reduced after employing the NFES-CPW.

**Conclusions:**

Therefore, NFES-CPW is an appropriate tool that adheres to Chinese dietary characteristics and can provide suitable dietary guidance to pregnant women.

**Supplementary Information:**

The online version contains supplementary material available at 10.1186/s12937-023-00902-4.

## Introduction

The maternal dietary nutrition status throughout pregnancy has long-term consequences on maternal and offspring health [1]. Maternal gestational diabetes mellitus (GDM), gestational hypertension, intrauterine growth restriction of the fetus, abnormal neurocognitive development, congenital defects [2], macrosomia [3], obesity [4], and offspring allergy [5] can all be caused by inadequate or excessive nutrition during pregnancy. Therefore, suitable dietary guidance throughout pregnancy is important to ensure a balanced nutrient intake for pregnant women and sustain the mother’s and offspring’s health [6].

The food exchange system is one of the most commonly utilized dietary guidance methods, and its first edition was published in America in 1950 [7]. Up to know, the American exchange lists have been modified several times for dietary nutrition guidance for various physiologic [8–10] and/or pathologic conditions, such as for patients with chronic diseases [11], vegetarians [12], and overweight or obese individuals [13], however, pregnancy has not been included. A study in Spain examined the nutrients that are particularly susceptible to lack during pregnancy, such as iron and calcium, when designing a food exchange system, in addition to carbohydrates [11]. This is a great start, although the attempts made are limited.

The traditional food exchange systems, which are mainly adopted in China, were designed for the diet management of patients rather than healthy people, considering energy the only basis for food exchange [14]. It is currently utilized in the diet management of pregnant women with overweight or obese [15] or those with gestational diabetes [16]. However, in addition to pregnant women with special diseases, China has thousands of healthy pregnant women. It is also critical to ensure that they have a balanced dietary nutrition supply during pregnancy to protect the health of both mothers and infants. Focusing on energy is insufficient for these pregnant women.

The body needs more energy and nutrients during pregnancy [17]. Currently, the nutritional issue of Chinese pregnant women that deserves special attention is excessive energy intake with a deficiency of micronutrients such as protein, calcium, iron, zinc, iodine, vitamin A (VA), vitamin B_1_ (VB_1_), vitamin B_2_ (VB_2_), vitamin B_12_ (VB_12_), and folic acid [18]. Therefore, dietary guidance that does not account for nutrient imbalances is insufficient. A particular food exchange system for pregnant women is required. While we must ensure that our calorie consumption is not excessive, we must also examine the dietary adequacy of other key nutrients. Simultaneously, the limited variety of food and portion size in the traditional food exchange systems were not equivalent to the commonly used household measures [14].

This study aimed to design a novel food exchange system for Chinese pregnant women (NFES-CPW) based on Chinese traditional dishes, which covered food types from all categories and had elaborate portion sizes and nutrients crucial for pregnant women. Moreover, we investigated the relative validity of the NFES-CPW by comparing the NFES-CPW to the traditional food exchange system when used to generate recipes.

## Methods

### Development of NFES-CPW

**Fig. 1:**
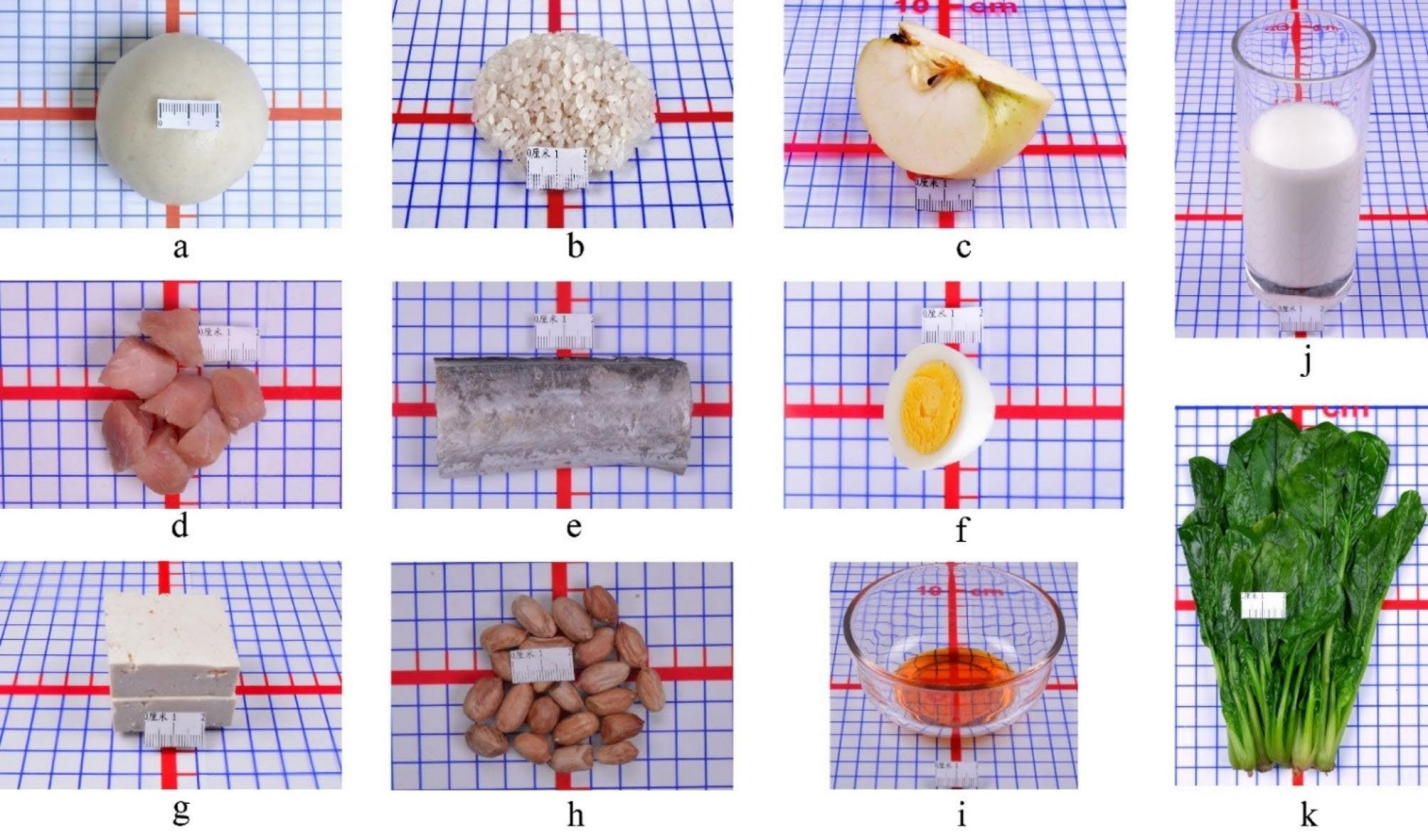
The representative food of each category (one portion size) **a**, steamed bread made of 25 g of wheat flour; **b**, 25 g of dry rice; **c**, 50 g of apple; **d**, 25 g of fresh lean pork; **e**, 25 g of fresh hairtail; **f**, 25 g of fresh whole chicken eggs; **g**, tofu made of 15 g of dry soybeans; **h**, 15 g of fried peanuts; **i**, 10 g of peanut oil; **j**, 125 g of fresh whole cow’s milk; **k**, 50 g of fresh spinach

As shown in Table 1, based on “*China Food Composition Table * [19]” and “*2016 Chinese Balanced Dietary Pagoda for pregnant women* [20]”, a total of 500 food types in the Chinese diet were summarized and classified into ten categories. Based on the main nutritional contribution of each food category, one or two exchange bases, such as energy, dry matter, protein were selected, and the specific values of the determined exchange bases were then arranged in ascending order. The food items with values close to the median and common in daily diet were considered representative foods (Fig. 1). The portion size of the representative food of each food category was determined based on the use in daily cooking, such as 25 or 50 g was equivalent to one portion size. Other food items in the same category were also assigned a portion size that had an equivalent exchange basis with the portion size of the representative food.

**Table 1:** Food categories and characteristics in the NFES-CPW

Food categories	Food exchange bases	Representative food	Portion size
Cereal and its products, potatoes, and beans excluding soybeans	energy	dry rice or wheat flour	25 g (86 kcal energy)
Vegetables	energy and dry matter	fresh spinach	50 g (12.5 kcal energy and water content accounts for 90% of total weight)
Fruits	energy and dry matter	apple	50 g (25 kcal energy and water content accounts for 85% of total weight)
Livestock and poultry meat	energy and protein	fresh lean pork	25 g (70 kcal energy and 10 g protein)
Fish, shrimp and shellfish	energy and protein	fresh hairtail	25 g (27.5 kcal energy and 4.5 g protein)
Eggs	energy and protein	fresh whole chicken eggs	25 g (35 kcal energy and 3 g protein)
Milk and its products	energy and protein	fresh whole cow’s milk	125 g (70 kcal energy and 4 g protein)
Soybean and its products	energy and protein	dry soybeans	15 g (60 kcal energy and 6 g protein)
Nuts	energy and protein	fried peanuts	15 g (90 kcal energy and 4 g protein)
Cooking oils	energy	peanut oil	10 g (90 kcal energy)

NFES-CPW, Novel food exchange system for Chinese pregnant women

To provide effective guidance to non-professionals and encourage users to select food items with rich nutrition density, the recommendation index was set at 0 − 5 from the perspective of nutrition professionals [19]. A recommendation index of five indicated the most recommended food whereas zero indicated the least recommended food (Supplementary Table 1 for details).

### Relative validity of NFES-CPW

#### Recruitment of dietitians and derivation of recipes

The growth and development of the fetus are relatively slow in the first trimester, and the nutrition required is not significantly different from that before pregnancy. However, beginning in the second trimester, the changes in the fetus and the mother accelerate, and the nutritional needs rise. Therefore, combined with the article’s length limitation, evaluation of the relative validity is limited to the second trimester of pregnancy in this study. A sample one-day recipe for women in their second trimester of pregnancy was developed by us based on the recommended food intake in the “*2016 Chinese Balanced Dietary Pagoda for pregnant women* [20]” and the reference intake of one-day energy and nutrients of pregnant women in the “*2013 Chinese Dietary Reference Intakes (DRIs)* [21]”. The food information obtained from the sample recipe was shown in Supplementary Table 2.

E-mail invitations were sent to the dietitians from the Nutrition Department of a third-level first-class hospital. They were required to have a bachelor’s degree or higher in their major, have worked in dietary management in the Nutrition Department of the hospital for > 3 years, and have clinician or dietitian qualifications. Finally, ten dietitians were recruited, and detailed documents containing recipe samples and NFES-CPW were delivered. In the second trimester of pregnancy, each dietitian was instructed to use the NFES-CPW and traditional food exchange system to derive four new recipes from the sample recipes. There was a standard of creating the recipes and all dietitians received technical training. Traditional food exchange system relies on energy as the basis for food exchange. When using NFES-CPW, the new recipes were created according to the nutritional characteristics of each food category, and the exchange bases are detailed in Table 1. There were 80 derived recipes in total, 40 for each of the two food exchange methods.

#### Assessment of dietary information

In the derived recipes, the total daily consumption of each type of food was calculated. Following the calculation of the total daily intake of each food group, the proportion of recipes that meet the recommended intake of each food category is determined using the “*2016 Chinese Balanced Dietary Pagoda for pregnant women* [20]”. The energy and nutritional content of each food were calculated using the “*China Food Composition Table * [19]”. An excel worksheet was used for food conversion and calculation. Carbohydrates, fats, and proteins were converted from g to kcal (1 g fat = 9 kcal; 1 g carbohydrate and protein = 4 kcal) to calculate the contribution of these macronutrients to the energy content of the food. The energy and nutrients delivered by the derived recipes were analyzed using the “*2013 Chinese DRIs* [21]”. The energy measurements were calculated using the estimated energy reference (EER) value. The intake of most nutrients was calculated using the estimated average reference (EAR) and recommended nutrient intake (RNI), including carbohydrate, protein, VA, VB_1_, VB_2_, folic acid, VB_12_, vitamin C (VC), calcium, iron, zinc, and iodine. Adequate intake (AI) was used to evaluate insoluble dietary fiber. Furthermore, for carbohydrates and fats, the acceptable macronutrient distribution ranges (AMDR) were used as the lower and upper bounds for the percentage ratio of energy.

### Data analysis

After assessing the normality of the continuous variables, we observed that all continuous variables were non-normally distributed (*P* < 0.05) and expressed in quartiles; median (*P*_25_; *P*_75_). Categorical variables were expressed as frequency (n) and percentage (%). The Wilcoxon rank sum test and the Chi-square (χ²) test were employed to compare the differences in food, energy, and nutrient intake of the new recipes derived from two exchange systems and those in the proportion of these intakes within the recommended range, respectively.

Energy and nutrient intake of the new recipes derived from the traditional food exchange system or NFES-CPW were compared with those derived from the sample recipes. The relative difference (d), the percentage of d (d%), the absolute difference (D), and the percentage of D (D%) were calculated:

d = data in the derived recipe - data in the sample recipe;

D = |data in the derived recipe - data in the sample recipe|;

d% = (d/data in the sample recipe) ×100%;

D% = (D/data in the sample recipe) ×100%.

The Wilcoxon rank sum test was used to analyze the differences in d, d%, D, and D% determined among the new recipes derived from two food exchange systems.

All statistical tests were two-sided, and *P* < 0.05 was considered statistically significant. All statistical analyses of this study were performed using the Statistical Package for the Social Sciences version 26.0 (IBM, New York, NY, USA).

## Results

### Comparison between food intakes of different categories in derived recipes

As presented in Table 2, the intake of cereal and its products, potatoes, and beans excluding soybeans from the new recipes derived from the NFES-CPW was significantly higher than that derived from the traditional food exchange system. This increase was mainly reflected in an increase in the consumption of whole-grain cereals and beans excluding soybeans, and potatoes, which significantly increased the proportion of these two food items within the recommended range by 90% and 77.5%, respectively, compared to the traditional food exchange system.

A similar result was observed in the intake of livestock and poultry meat, fish, shrimp, shellfish, and eggs. The intake of this food category was considerably higher in the new recipes derived from the NFES-CPW than that in the traditional food exchange system. This increase was mainly reflected in an increase in the intake of fish, shrimp, shellfish, and eggs, which leads to a significantly higher proportion of these two food items in the recommended range than that obtained by the traditional food exchange system, with increases of 62.5% and 25%, respectively.

There was no statistical difference in the intake of vegetables and milk and its products among the new recipes obtained from the two food exchange systems, and the intakes of vegetables (100%) and milk and its products (97.5%) in almost all recipes were within the recommended range. Based on their low total intake, the intakes of soybean and its products and nuts in the new recipes obtained from the two food exchange systems were not significantly different.

The intake of fruit in the new recipes derived from the traditional food exchange system was at a high level, and the proportion of its intake in the recommended range was 51.5%. However, the intake of fruit in the derived recipes based on NFES-CPW decreased significantly, thus, increasing its proportion in the recommended range to 97.5%.

**Table 2:** Comparison of food intake (g/d) in recipes within the recommended range in Dietary Pagoda

	Traditional		NFES-CPW	
	Median (*P*_25_, *P*_75_)	Rec [n (%)]	Median (*P*_25_, *P*_75_)	Rec [n (%)]
Cereal and its products, potatoes, and beans excluding soybeans	309.5 (297.0, 318.8)	29 (72.5)	324.0 (319.0, 349.0)^**^	23 (57.5)
Whole-grain cereals, and beans excluding soybeans	35.0 (0.0, 40.0)	0 (0.0)	77.0 (75.0, 81.5)^**^	36 (90.0)^**^
Potatoes	50.0 (50.0, 50.0)	9 (22.5)	75.0 (75.0, 97.0)^**^	40 (100.0)^**^
Vegetables	405.0 (382.5, 430.5)	40 (100.0)	388.0 (367.5, 423.8)	40 (100.0)
Fruits	394.5 (345.3, 440.5)	21 (52.5)	365.0 (339.3, 379.5)^**^	39 (97.5)^**^
Livestock and poultry meat, fish, shrimp and shellfish, and eggs	151.5 (135.5, 166.3)	22 (55.0)	180.0 (175.0, 190.0)^**^	37 (92.5)^**^
Livestock and poultry meat	70.0 (54.0, 84.5)	21 (52.5)	70.0 (70.0, 78.0)	29 (72.5)
Fish, shrimp and shellfish	32.0 (0.0, 60.0)	11 (27.5)	60.0 (50.0, 70.0)^**^	36 (90.0)^**^
Eggs	50.0 (50.0, 50.0)	29 (72.5)	50.0 (50.0, 50.0)	39 (97.5)^**^
Milk and its products	420.0 (388.0, 431.8)	39 (97.5)	420.0 (398.0, 420.0)	39 (97.5)
Soybean and its products	16.0 (16.0, 18.0)	0 (0.0)	15.5 (14.3, 16.0)^**^	0 (0.0)
Nuts	10.0 (10.0, 10.0)	37 (92.5)	10.0 (9.0, 10.0)	20 (50.0)^**^

NFES-CPW, Novel food exchange system for Chinese pregnant women; Rec, The number and percentage of derived recipes with food consumption within the recommended range

*P*-values were assessed using the Wilcoxon rank sum test for non-normally distributed continuous variables or χ² test for categorical variables; ^ns^*P* > 0.05, non-significant; ^*^P < 0.05, significant; ^**^P < 0.01, significant

**Table 3:** Comparison between energy and nutrient intakes in recipes with Chinese dietary reference intakes

	Traditional			NFES-CPW		
	Median (*P*_25_, *P*_75_)	Below EER/EAR [n (%)]	Above RNI/AI [n (%)]	Median (*P*_25_, *P*_75_)	Below EER/EAR [n (%)]	Above RNI/AI [n (%)]
Energy (kcal/d)	2204.4 (2154.9, 2262.1)	1 (2.5)	/	2177.5 (2134.5, 2218.0)^**^	3 (7.5)	/
Carbohydrate (g/d)	316.2 (308.4, 327.5)	0 (0.0)	/	308.6 (296.7, 321.3)^*^	0 (0.0)	/
Fat (g/d)	72.9 (67.1, 81.3)	/	/	70.6 (67.2, 73.5)^*^	/	/
Protein (g/d)	78.3 (76.0, 79.5)	0 (0.0)	40 (100.0)	84.9 (82.3, 88.8)^**^	0 (0.0)	40 (100.0)
Insoluble dietary fiber (g/d)	16.0 (13.8, 19.3)	/	0 (0.0)	20.3 (17.0, 21.4)^**^	/	2 (5.0)
VA (µg RAE/d)	524.2 (467.7, 589.2)	21 (52.5)	1 (2.5)	853.0 (799.2, 1003.3)^**^	0 (0.0)**	37 (92.5)^**^
VB_1_ (mg/d)	1.1 (1.0, 1.2)	10 (25.0)	0 (0.0)	1.2 (1.1, 1.3)^**^	4 (10.0)	0 (0.0)
VB_2_ (mg/d)	1.4 (1.2, 1.5)	0 (0.0)	11 (27.5)	1.6 (1.4, 1.8)^**^	0 (0.0)	26 (65.0)^**^
Folic acid (µg DFE/d)	323.1 (273.7, 382.1)	40 (100.0)	0 (0.0)	632.5 (620.9, 674.2)^**^	0 (0.0)**	38 (95.0)^**^
VB_12_ (µg/d)	2.3 (1.3, 8.6)	20 (50.0)	15 (37.5)	10.2 (1.9, 17.3)^**^	12 (30.0)	25 (62.5)^*^
VC (mg/d)	107.2 (93.5, 118.4)	10 (25.0)	14 (35.0)	146.6 (133.7, 161.8)^**^	0 (0.0)**	40 (100.0)^**^
Calcium (mg/d)	913.1 (870.8, 958.30)	5 (12.5)	1 (2.5)	1163.6 (1107.1, 1302.8)^**^	0 (0.0)	39 (97.5)^**^
Iron (mg/d)	19.1 (17.8, 21.6)	19 (47.5)	7 (17.5)	27.2 (25.2, 31.9)^**^	0 (0.0)**	32 (80.0)^**^
Zinc (mg/d)	11.0 (10.4, 12.1)	0 (0.0)	38 (95.0)	14.0 (12.8, 14.6)^**^	0 (0.0)	40 (100.0)
Iodine (µg/d)	193.2 (166.4, 201.8)	5 (12.5)	2 (5.0)	241.0 (232.5, 261.0)^**^	1 (2.5)	36 (90.0)^**^

NFES-CPW, Novel food exchange system for Chinese pregnant women; EER, estimated energy requirement; EAR, estimated average requirement; RNI, recommended nutrient intake; AI, adequate intake; RAE, retinol activity equivalents; DFE, dietary folate equivalent

Below EER/EAR: The number and percentage of derived recipes with energy or nutrient intakes below the EER or EAR value

Above RNI/AI: The number and percentage of derived recipes with nutrient intakes above the RNI or AI value

*P*-values were assessed using the Wilcoxon rank sum test for non-normally distributed continuous variables or χ² test for categorical variables; ^ns^*P* > 0.05, non-significant; ^*^P < 0.05, significant; ^**^P < 0.01, significant

### Comparison between energy and nutrient intakes in derived recipe

As presented in Table 3, compared with the recipes derived from the traditional food exchange system, the energy intake of those derived from NFES-CPW decreased, accompanied by a decrease in carbohydrate and fat intake and an increase in protein intake. The difference between the two food exchange systems was statistically significant. Further, the contribution of carbohydrate, fat, and protein intake to total energy intake was calculated (Table 4). Only protein, albeit not a carbohydrate and fat, exhibited a statistically significant difference in the contribution to total energy intake among the two exchange systems (*P* < 0.05). When compared with the relevant indicators in the “*2013 Chinese DRIs* [21]”, it was observed that the energy, carbohydrate, and protein intakes in the recipes derived from the two food exchange systems were suitable. However, the proportion of the contribution of fat intake to total energy intake in the AMDR range was higher in the recipes derived from NFES-CPW (60%) than those derived from the traditional food exchange system (45%), however, the difference was not statistically significant.

Insoluble dietary fiber and representative vitamins (VA, VB_1_, VB_2_, folic acid, VB_12_, and VC) and minerals (calcium, iron, zinc, and iodine) were selected for further investigation based on the nutritional needs of women during pregnancy. As presented in Table 3, compared with those in the recipes derived from the traditional food exchange system, the intake of all these nutrients in those derived from the NFES-CPW statistically increased. When compared with the relevant indicators in the “*2013 Chinese DRIs* [21]”, we observed that the proportion of the derived recipes derived from NFES-CPW with the intakes of VA, folic acid, VC, and iron below the EAR value was lower than that derived from traditional food exchange system (*P* ≤ 0.01). Meanwhile, the proportion of the recipes derived from NFES-CPW with the intakes of VA, VB_2_, folic acid, VB_12_, VC, calcium, iron, and iodine above the RNI value was higher than that derived from the traditional food exchange system (*P* < 0.05).

**Table 4:** Comparison of the contribution of carbohydrate, fat, and protein intakes to the total energy intake in recipes

Energy ratio (%E)	Traditional		NFES-CPW	
	Median (*P*_25_, *P*_75_)	Within AMDR [n (%)]	Median (*P*_25_, *P*_75_)	Within AMDR [n (%)]
Carbohydrate	57.6 (54.9, 59.1)	40 (100.0)	56.4 (55.3, 58.6)	40 (100.0)
Fat	30.1 (28.0, 32.5)	18 (45.0)	29.4 (27.4, 30.6)	24 (60.0)
Protein	14.1 (13.7, 14.6)	-	15.7 (15.1, 16.5)^**^	-

NFES-CPW, Novel food exchange system for Chinese pregnant women; AMDR, acceptable macronutrient distribution ranges

Within AMDR: The number and percentage of derived recipes with the intake of energy ratio provided by carbohydrates or fat within the AMDR.

*P*-values were assessed using the Wilcoxon rank sum test for non-normally distributed continuous variables or χ² test for categorical variables; ^ns^*P* > 0.05, non-significant; ^*^P < 0.05, significant; ^**^P < 0.01, significant

### Differences in energy and nutrient intakes between derived recipes and sample recipes

Compared with the traditional food exchange system, the median values of energy and nutrient intakes in the recipes derived from NFES-CPW were closer to their quantities in the sample recipes. The difference between the two food exchange systems was statistically significant.

As presented in Table 5, a further calculation was performed. Compared with the sample recipes, in the new recipes derived from the traditional food exchange system, the median d-value of all nutrients except energy, carbohydrate, and fat was < 0. After using the NFES-CPW, the d-value decreased and became positive, indicating that the rectification of nutrients using NFES-CPW was directional.

The absolute value was further calculated, and it was observed that, except for carbohydrates and zinc, the median D-value of energy and nutrients in the recipes derived from NFES-CPW was lower than that derived from the traditional food exchange system. Significant differences were observed in protein, fat, insoluble dietary fiber, VA, VB_1_, folic acid, VB_12_, VC, calcium, iron, and iodine (*P* < 0.05), indicating that the intake of these nutrients in recipes derived from NFES-CPW was close to their quantities in the sample recipes. Compared with the recipes derived from the traditional food exchange system, after using the NFES-CPW, the error ranges of these nutrients were reduced by 5.3% (protein), 14% (insoluble dietary fiber), 28.3% (VA), 7.7% (VB_1_), 45% (folic acid), 40.1% (VB_12_), 18.8% (VC), 17.3% (calcium), 23.3% (iron), and 9.6% (iodine).

**Table 5:** Comparison between energy and nutrients in derived recipes with the sample recipe

	Sample recipe	Derived recipes	Median (*P*_25_, *P*_75_)	d	d%	D	D%
Energy (kcal/d)	2185.1	Traditional	2204.4 (2154.9, 2262.1)	19.3 (-30.3, 77.0)	0.9 (-1.4, 3.5)	48.5 (25.1, 90.5)	2.2 (1.1, 4.1)
		NFES-CPW	2177.5 (2134.5, 2218.0)^**^	-7.7 (-50.6, 32.9)^**^	-0.4 (-2.3, 1.5)^**^	37.8 (16.8, 66.5)	1.7 (0.8, 3.0)
Protein (g/d)	86.1	Traditional	78.3 (76.0, 79.5)	-7.8 (-10.1, -6.7)	-9.1 (-11.7, -7.7)	7.8 (6.7, 10.1)	9.1 (7.7, 11.7)
		NFES-CPW	84.9 (82.3, 88.8)^**^	-1.2 (-3.8, 2.7)^**^	-1.4 (-4.4, 3.2)^**^	3.3 (1.9, 5.1)^**^	3.8 (2.1, 5.9)^**^
Carbohydrate (g/d)	311.9	Traditional	316.2 (308.4, 327.5)	4.3 (-3.5, 15.6)	1.4 (-1.1, 5.0)	8.7 (3.4, 16.7)	2.8 (1.1, 5.4)
		NFES-CPW	308.6 (296.7, 321.3)^*^	-3.3 (-15.2, 9.4)^*^	-1.1 (-4.9, 3.0)^*^	13.4 (7.3, 22.5)	4.3 (2.3, 7.2)
Fat (g/d)	71.5	Traditional	72.9 (67.1, 81.3)	1.4 (-4.4, 9.8)	2 (-6.1, 13.7)	5.6 (-2.2, 10.2)	7.8 (3.1, 14.3)
		NFES-CPW	70.6 (67.2, 73.5)^*^	-1.0 (-4.3, 2.0)^*^	-1.3 (-6.0, 2.8)^*^	2.9 (1.3, 4.8)^**^	4.0 (1.8, 6.7)
Insoluble dietary fiber (g/d)	21.0	Traditional	16 (13.8, 19.3)	-5.0 (-7.2, -1.8)	-23.8 (-34.2, -8.3)	5.0 (2.2, 7.2)	23.8 (10.5, 34.2)
		NFES-CPW	20.3 (17.0, 21.4)^**^	-0.8 (-4.0, 0.4)^**^	-3.6 (-18.9, 1.8)^**^	2.1 (0.6, 4.6)^**^	9.8 (2.6, 21.9)^**^
VA (µg RAE/d)	1144.0	Traditional	524.2 (467.7, 589.2)	-619.9 (-676.3, -554.8)	-54.2 (-59.1, -48.5)	619.9 (554.8, 676.3)	54.2 (48.5, 59.1)
		NFES-CPW	853.0 (799.2, 1003.3)^**^	-291.0 (-344.8, -140.7)^**^	-25.4 (-30.1, -12.3)^**^	296.1 (181.6, 346.7)^**^	25.9 (15.9, 30.3)^**^
VB_1_ (mg/d)	1.3	Traditional	1.1 (1.0, 1.2)	-0.2 (-0.3, -0.1)	-15.4 (-21.2, -7.7)	0.2 (0.1, 0.3)	15.4 (7.7, 21.2)
		NFES-CPW	1.2 (1.1, 1.3)^**^	-0.1 (-0.2, 0.0)^**^	-7.7 (-15.4, 0.0)^**^	0.1 (0.1, 0.2)^**^	7.7 (7.7, 15.4)^**^
VB_2_ (mg/d)	1.6	Traditional	1.4 (1.2, 1.5)	-0.2 (-0.4, -0.1)	-12.5 (-25.0, -6.3)	0.2 (0.1, 0.4)	12.5 (7.8, 25.0)
		NFES-CPW	1.6 (1.4, 1.8)^**^	-0.1 (-0.2, 0.2)^**^	-3.1 (-12.5, 10.9)^**^	0.2 (0.1, 0.3)	12.5 (6.3, 18.8)
Folic acid (µg DFE/d)	626.3	Traditional	323.1 (273.7, 382.1)	-303.2 (-352.6, -244.2)	-48.4 (-56.3, -39.0)	303.2 (244.2, 352.6)	48.4 (39.0, 56.3)
		NFES-CPW	632.5 (620.9, 674.2)^**^	6.2 (-5.4, 47.9)^**^	1.0 (-0.9, 7.6)^**^	21.3 (5.3, 48.9)^**^	3.4 (0.8, 7.8)^**^
VB_12_ (µg/d)	18.1	Traditional	2.3 (1.3, 8.6)	-15.9 (-16.8, -9.5)	-87.6 (-92.7, -52.6)	15.9 (9.5, 16.8)	87.6 (52.6, 92.7)
		NFES-CPW	10.2 (1.9, 17.3)^**^	-8.0 (-16.2, -0.8)^**^	-43.9 (-89.5, -4.6)^**^	8.6 (3.6, 16.2)^**^	47.5 (19.8, 89.5)^**^
VC (mg/d)	145.6	Traditional	107.2 (93.5, 118.4)	-38.5 (-52.2, -27.2)	-26.4 (-35.8, -18.7)	40.2 (29.2, 53.6)	27.6 (20.1, 36.8)
		NFES-CPW	146.6 (133.7, 161.8)^**^	1.0 (-11.9, 16.2)^**^	0.7 (-8.2, 11.1)^**^	12.8 (6.6, 23.0)^**^	8.8 (4.5, 15.8)^**^
Calcium (mg/d)	1295.8	Traditional	913.1 (870.8, 958.3)	-382.8 (-425.0, -337.5)	-29.5 (-32.8, -26.0)	382.8 (337.5, 425.0)	29.5 (26.0, 32.8)
		NFES-CPW	1163.6 (1107.1, 1302.8)^**^	-132.3 (-188.7, 7.0)^**^	-10.2 (-14.6, 0.5)^**^	157.5 (91.7, 208.9)^**^	12.2 (7.1, 16.1)^**^
Iron (mg/d)	29.8	Traditional	19.1 (17.8, 21.6)	-10.7 (-12.1, -8.2)	-35.9 (-40.4, -27.4)	10.7 (8.2, 12.1)	35.9 (27.4, 40.4)
		NFES-CPW	27.2 (25.2, 31.9)**	-2.6 (-4.7, 2.1)**	-8.7 (-15.6, 7.0)**	3.8 (2.2, 6.7)**	12.6 (7.3, 22.4)**
Zinc (mg/d)	12.2	Traditional	11.0 (10.4, 12.1)	-1.3 (-1.8, -0.1)	-10.2 (-14.8, -0.8)	1.4 (0.9, 1.9)	11.5 (7.0, 15.6)
		NFES-CPW	14.0 (12.8, 14.6)**	1.8 (0.6, 2.4)**	14.8 (5.1, 19.7)**	1.8 (0.9, 2.4)	14.8 (7.0, 19.7)
Iodine (µg/d)	229.2	Traditional	193.2 (166.4, 201.8)	-36.0 (-62.8, -27.4)	-15.7 (-27.4, -12.0)	36.0 (27.4, 62.8)	15.7 (12.0, 27.4)
		NFES-CPW	241.0 (232.5, 261.0)**	11.8 (3.3, 31.8)**	5.1 (1.5, 13.9)**	14.0 (6.4, 37.0)**	6.1 (2.8, 16.2)**

NFES-CPW, Novel food exchange system for Chinese pregnant women; RAE, retinol activity equivalents; DFE, dietary folate equivalent; d, the relative difference; D, the absolute difference

d = data in the derived recipe - data in the sample recipe; D = |data in the derived recipe - data in the sample recipe|; d% = (d/data in the sample recipe) ×100%; D% = (D/data in the sample recipe) ×100%

*P*-values were assessed using the Wilcoxon rank sum test for non-normally distributed continuous variables; ^ns^*P* > 0.05, non-significant; ^*^P < 0.05, significant; ^**^P < 0.01, significant

## Discussion

A healthy and diverse diet remains the preferred means to address the nutritional requirements of pregnant women [22]. The food exchange system is a simple and practical method for incorporating abstract food exchange and a sophisticated balanced diet into daily life. In contrast to the previous studies [23, 24], the food exchange system developed and validated in our study, called NFES-CPW, was particularly designed for healthy pregnant women in China, not pregnant women with gestational diabetes or those who were overweight or obese. When compared with traditional food exchange systems, NFES-CPW has the advantages of covering more food types from all categories, elaborating food portion sizes, guiding food selection, and regulating the energy and key nutrient supply during pregnancy.

To ensure adequate energy and nutrient intake by pregnant women, NFES-CPW determines the food exchange bases according to the nutritional characteristics of each food category and not just energy. Cereal and their products, potatoes, and beans excluding soybeans were the major sources of the total dietary energy supply [25, 26]. Additionally, the main component of cooking oil was fat, which provides energy, and this cannot be overlooked [27]. Thus, energy serves as the foundation for these two food categories in the NFES-CPW, similar to how it does for traditional food exchanges. Vegetables and fruits mainly provide dietary fibers, vitamins, and minerals [28]. The richness of these nutrients is represented by the dry matter content in these two food categories, and the water content and dry matter content are complementary [29]. Therefore, in contrast to the traditional food exchange system, the water content was selected as one of the food exchange bases of vegetables and fruits in NFES-CPW. The biggest difference between animal food categories (including livestock and poultry meat, fish, shrimp and shellfish, eggs, and milk and its products) and plant food categories is that the animal food categories can provide high-quality protein [30]. Therefore, besides energy, NFES-CPW selected protein as one of the food exchange bases for these food categories. It is noteworthy that, since livestock and poultry meat varies greatly in terms of its fat content [31], different weights of protein and energy were assigned to the various classifications in the NFES-CPW after the meat was categorized according to its fat content and energy contribution from fat. Among plant food categories, soybean and its products and nuts were considered good sources of fat and protein; thus, in NFES-CPW, energy and protein were selected as food exchange bases for these two food categories [32, 33].

This study simulates the use of the NFES-CPW and traditional food exchange system by dietitians to derive new recipes based on the sample recipe, similar to the use of the food exchange system in real life. The results revealed that the recipes derived from NFES-CPW had better dietary structures compared with those derived from traditional food exchange system. It was primarily reflected in the fact that the proportion of whole-grain cereals, beans excluding soybeans, potatoes, fruits, fish, shrimp and shellfish, and egg intakes within the recommended range in the recipes derived from NFES-CPW were significantly higher than those derived from the traditional food exchange system. However, it is noteworthy that the proportion of nut intake within the recommended range in recipes derived from NFES-CPW was 42.5% less than that in recipes derived from the traditional food exchange system. Considering that nuts are rich in fat and energy, pregnant women should limit their intake [33]. The recommended index of nuts in NFES-CPW was set at four instead of five, which may affect the setting of nut intake in NFES-CPW derived recipes. Additionally, the total recommended intake of nuts is 10 g, which is a relatively small value and can easily fluctuate the results.

Energy balance was one of the key factors for achieving optimal pregnancy outcomes. Studies have demonstrated that a balanced intake of energy and protein during pregnancy can significantly reduce the risk of stillbirth, small-for-gestational-age, and macrosomia and maintain normal birth weight [34, 35]. Nevertheless, the energy contribution of macronutrients in most Chinese pregnant women was unbalanced, and the energy contribution from fat was excessive [18]. A previous study used a traditional food exchange system based on GI to provide dietary guidance to patients with GDM and demonstrated that the energy intake and postprandial blood glucose of patients were effectively controlled [23]. Another study used a traditional food exchange system to intervene in the diet of obese or overweight pregnant women, which effectively controlled the energy intake and weight gain during pregnancy in patients [24]. Similarly, in our study, the energy, carbohydrate, and protein intakes in the recipes derived from the two food exchange systems were suitable, which revealed that these two food exchange systems have similar effects on the dietary energy and macronutrient intake of pregnant women. Our study also demonstrated that the first advantage of employing NFES-CPW is that it brings the calorie, protein, and fat intakes in recipes closer to the amounts in the sample recipe, as opposed to those produced from typical food exchange systems.

In this study, vitamins were further analyzed. Folic acid deficiency in the first trimester can cause stillbirth, abortion [36], and neural tube malformation [37]. Therefore, most Chinese pregnant women are more concerned about folic acid intake in the first trimester but overlook it during the second trimester. A dietary survey conducted in eight cities in China revealed that the average folic acid intake by pregnant women was lower than the RNI level in all trimesters, and the trend of folic acid intake drastically decreased from the second trimester [18]. In fact, during the second trimester, the increase in blood volume is significantly greater than that in red blood cell count, and pregnant women are vulnerable to dilutional physiological anemia [38]. Folic acid deficiency affects the DNA synthesis in erythroblastic nuclei, causing megaloblastic anemia [39]. It can also cause homocysteinemia, which can induce hypertensive disorders in pregnancy [40]. VA is an important vitamin to maintain normal vision, immunity, reproduction, and embryonic development and growth [41]. The prevalence of VA deficiency (VAD) and marginal VAD in Chinese pregnant women were 1.2% and 10.5%, respectively [42]. Encouraging pregnant women to increase food consumption rich in preformed VA (retinol and retinyl ester) and provitamin A (beta-carotenoid) is the most lasting way to fundamentally improve the nutritional status of VA [43]. However, previous exchange systems have not focused on vitamin intake [11, 15, 24]. The results of this study revealed that dietary folic acid and VA levels were significantly improved by NFES-CPW, and the proportions of folic acid and VA intakes that reached the RNI levels were 95% and 90% higher than those of the traditional food exchange system, respectively, indicating the second advantage of NFES-CPW.

Additionally, important minerals for pregnant women include calcium and iron. During the second and third trimesters, calcium is gradually deposited in the fetal bones and teeth, and approximately 30 g of calcium is deposited in the newborn [44]. If calcium deficiency occurs during pregnancy, the mother will use the calcium deposited in her bones to maintain the blood calcium concentration and meet the needs of fetal bone growth and development [45]. Therefore, calcium intake during pregnancy will affect not only the fetal health but also the mother’s health. Studies have revealed that low dietary calcium intake during pregnancy was associated with the lower bone mineral density of mothers following parturition [45] and their offspring [46] and increased risks of hypertensive disorders during pregnancy [47] and mental health disorders in childhood [48, 49]. As aforementioned, in the second and third trimesters, pregnant women are susceptible to dilutional physiological anemia. Furthermore, the growth of fetal and placental tissues requires additional iron [50]. Therefore, if the dietary iron intake during pregnancy is inadequate, iron deficiency anemia or iron deficiency can easily occur in pregnant women and infants, which will cause placental hypoxia, reduce maternal immunity, and increase the risk of premature delivery, low birth weight of the offspring, and cognitive impairment in childhood [51]. However, the calcium and iron intakes of pregnant women in China are not encouraging. As presented in a study conducted in Sichuan Province, the proportions of calcium and iron intakes among pregnant women that reached the RNI level were only 50.2% and 53.4%, respectively [52]. In this study, the third advantage of NFES-CPW was to significantly increase the level of dietary calcium and iron. The proportions of calcium and iron intakes that reached the RNI level were 95% and 62.5% higher than those in the traditional food exchange system, respectively. A previous study on the food exchange system involved calcium and iron, albeit it focused more on to dietary recommendations and did not run further simulations to evaluate the application of the food exchange system as our study did [11].

Our study does, however, have certain limitations. First, the nutritional data in the “*China Food Composition Table * [19]” were incomplete, and the content of some nutrients was not measured or detected using the current techniques. Hence, the nutrient intake in the recipes, such as the insoluble dietary fiber content, may be lower than the actual value. Second, the NFES-CPW was merely simulated to provide dietary guidance in our study, and its practicality and effectiveness in the clinical setting were not tested, which must be addressed in further studies. Last, our study’s sample size was small, and it must be increased and extended to additional special populations, like children and the elderly, who also urgently require nutritional counseling.

## Conclusions

NFES-CPW helps to meet the requirements of pregnant women for dietary structure, energy, and key nutrients. It is a simple and effective tool that conforms to Chinese dietary norms and may provide reasonable dietary guidance to pregnant women. We recommend that maternity nutrition clinics employ the NFE-CPW to create flexible meal programs to assist expectant mothers. Furthermore, the NFE-CPW can also be a useful tool for nutritionists to conduct nutritional education and for pregnant women to manage their own diet.

### Electronic supplementary material

Below is the link to the electronic supplementary material.

Supplementary Material 1

## Data Availability

The datasets used and analysed during the current study are available from the corresponding author on reasonable request.

## Abbreviations

NFES-CPW: Novel food exchange system for Chinese pregnant women
GDM: Gestational diabetes mellitus
GI: Glucose index
VA: Vitamin A
VB_1_: Vitamin B_1_
VB_2_: Vitamin B_2_
VB_12_: Vitamin B_12_
DRIs: Dietary Reference Intakes
EER: Estimated energy reference
EAR: Estimated average reference
RNI: Recommended nutrient intake
VC: Vitamin C
AI: Adequate intake
AMDR: Acceptable macronutrient distribution ranges
χ^²^: Chi-square
