# Supplementary material for: Development and validation of a novel food exchange system for Chinese pregnant women
Source: Nutr J. 2023 Dec 1;22:65. doi: 10.1186/s12937-023-00902-4 (PMC10690967; doi:10.1186/s12937-023-00902-4)
Supplement: Supplementary file 1 — Supplementary Material 1 [file 12937_2023_902_MOESM1_ESM.docx]

# Supplementary Material

**Supplementary Table 1. The recommendation index of food in each category**

| Food categories | Recommendation index | Portion size | Food examples |
| --- | --- | --- | --- |
| Cereal and its products, potatoes, and beans excluding soybeans **^a^** | 5 | 28 | Red bean; Mung bean; Whole wheat flour |
|  |  | 25 | Millet; Barley grain; Oats; Black rice |
|  | 4 | 30 | Wheat noodles; Bread |
|  |  | 25 | Wheat flour; Rice; Glutinous rice |
|  |  | 20 | Wheat pancake; Biscuits |
|  | 2 | 110 | Potato |
|  |  | 85 | Sweet potato |
|  |  | 80 | Corn (fresh) |
|  | 0 | 55 | Rice cakes (glutinous rice flour, steamed) |
|  |  | 20 | Wheat dough stick (deep-fried); Spring roll; Mooncake |
| Vegetables **^b^** | 5 | 100 | Romaine lettuce; Cucumber |
|  |  | 95 | Kelp (fresh) |
|  |  | 70 | Balsam pear; Celery stem; Asparagus |
|  |  | 60 | Oilseed rape |
|  |  | 50 | Spinach; Kidney bean; Amaranth (green) |
|  | 3 | 95 | Calabash; Cabbage |
|  |  | 70 | [White](C:/Users/Administrator.USER-20190917RV/AppData/Local/youdao/dict/Application/8.9.6.0/resultui/html/index.html#/javascript:;) [radish](C:/Users/Administrator.USER-20190917RV/AppData/Local/youdao/dict/Application/8.9.6.0/resultui/html/index.html#/javascript:;); Bamboo shoot |
|  |  | 65 | Eggplant |
|  |  | 60 | Water bamboo; Cauliflower |
|  | 1 | 130 | Chinese wax gourd; Conomon |
|  |  | 80 | Mung bean sprouts; Ginger |
|  |  | 20 | Taro; Beetroot |
| Fruits **^c^** | 5 | 90 | Bayberry |
|  |  | 85 | Strawberry; Carambola |
|  |  | 70 | Plum; Apricot; Loquat |
|  |  | 60 | Grape; Ponkan; Tangerine; Cherry; Pineapple |
|  |  | 55 | Pear; Peach; Orange; Guava; Pitaya |
|  |  | 50 | Apple; Citrus; Kumquat; Olive; Mulberry |
|  |  | 45 | Kiwi fruit |
|  |  | 40 | Fig; Blueberry |
| Livestock and Poultry meat **^d^** | 5 | 50 | Porcine blood; Duck blood; Pork small intestine |
|  |  | 30 | Pork heart; Chicken gizzard |
|  |  | 25 | Pork (lean); Beef (lean); Mutton (lean); Chicken |
|  | 2 | 25 | Chicken wing; Chicken heart; Duck intestine |
|  | 1 | 22 | Pork large intestine |
|  |  | 20 | Pork sausage; Duck tongue |
|  | 0 | 15 | Pork(lean and fat); Chicken (fat) |
|  |  | 10 | Preserved sausage; Preserved pork |
| Fish, shrimp and shellfish | 5 | 45 | Octopus; Swan mussel; Clam |
|  |  | 40 | Escargots; Scallops (fresh); Mussel (fresh) |
|  |  | 35 | Prawn; Multicolored abalone (marine); Stone snail |
|  |  | 30 | Shrimp; Squid; Cuttlefish |
|  |  | 25 | Yellow croaker (small); Hairtail; Codfish |
| Eggs | 5 | 25 | Chicken egg; Duck egg; Quail egg |
| Milk and its products | 5 | 125 | Cow milk; Yogurt |
|  |  | 20 | Cow milk powder; Cheese; Milk powder flakes |
| Soybeans and its products | 5 | 300 | Soybean milk |
|  |  | 90 | Soybean curd |
|  |  | 30 | Soybean curd sheet (rolled); Soybean curd slab |
|  |  | 15 | Soybean; Black soybean |
| Nuts **^e^** | 4 | 50 | Chestnut (cooked) |
|  |  | 20 | Walnut; Pine nut |
|  |  | 18 | White sesame; Black sesame |
|  |  | 15 | Almond kernel; Peanut kernel; Sunflower seed kernel |
| Cooking oils | 5 | 10 | Peanut oil; Corn oil; Rapeseed oil; Olive oil |

**^a^**: The recommendation index of this food category was 5−0, with five for whole-grain and coarse cereals, four for refined cereals, two for potatoes, and zero for starchy foods, desserts, and fried staples.

**^b^**: The recommendation index of vegetables was set based on their color, with five for dark-colored vegetables, three for light-colored vegetables, and one for white vegetables.

c: In the selection of fruit types, we considered glycemic index.

**^d^**: The weights of the food exchange bases vary between classifications, and the recommendation index was set at 5−0, respectively. For example, “lean meat” has an energy weight of 1/3, protein weight of 2/3, and recommendation index of five; however, “very greasy meat” has an energy weight of 9/10, protein weight of 1/10, and recommendation index of zero.

**^e^**: Since most nut types are rich in energy, pregnant women were required to limit their consumption, thus, the recommendation index of nuts was set at four.

**Supplementary Table 2. The food information obtained from the one-day sample recipe used in this study**

| Food categories | Recommended range^*^ (g) | Food items | Food weights (g) |
| --- | --- | --- | --- |
| Cereal and its products, potatoes, and beans excluding soybeans | 275-325 | Glutinous rice | 40 |
|  |  | Oats | 40 |
|  |  | Rice | 70 |
|  |  | Millet | 35 |
|  |  | Wheat noodles | 80 (= 67g Wheat flour) |
|  |  | Sweet potato | 75 |
| Vegetables | 300-500 | Oilseed rape | 80 |
|  |  | Spinach | 40 |
|  |  | Celery stalk | 90 |
|  |  | Zucchini | 60 |
|  |  | Tomato | 60 |
|  |  | Carrot | 20 |
|  |  | Kelp | 20 |
|  |  | Black fungus (soaked) | 20 |
|  |  | Shiitake mushroom | 30 |
| Fruits | 200-400 | Strawberry | 80 |
|  |  | Tangerine | 100 |
|  |  | Pitaya | 100 |
|  |  | Blueberry | 100 |
| Livestock and poultry meat | 50-75 | Pork (lean) | 40 |
|  |  | Duck liver | 30 |
| Fish, shrimp and shellfish | 50-75 | Hairtail | 50 |
| Eggs | 50 | Egg | 50 |
| Milk and its products | 300-500 | Yogurt | 200 |
|  |  | Milk | 250 |
| Soybean and its products | 20 | Soybean curd | 90 (= 17g dry soybean) |
| Nuts | 10 | Walnut kernels | 10 |
| Cooking oils | 25-30 | Sunflower seed oil | 29 |

^*^ Recommended intake of food for women in the second trimester of pregnancy from Chinese Balanced Dietary Pagoda
